# Functional activity changes after vergence and accommodative rehabilitation of concussion-related convergence insufficiency: CONCUSS clinical trial fMRI results

**DOI:** 10.3389/fnins.2025.1703781

**Published:** 2025-10-21

**Authors:** Ayushi Sangoi, Farzin Hajebrahimi, Suril Gohel, Mitchell Scheiman, Arlene Goodman, Melissa Noble, Tara L. Alvarez

**Affiliations:** ^1^Department of Biomedical Engineering, New Jersey Institute of Technology, Newark, NJ, United States; ^2^Department of Health Informatics, Rutgers University School of Health Professions, Newark, NJ, United States; ^3^Pennsylvania College of Optometry, Drexel University, Philadelphia, PA, United States; ^4^Somerset Pediatric Group, Raritan, NJ, United States; ^5^Comprehensive Sports Medicine & Concussion Care, LLC., Bridgewater, NJ, United States

**Keywords:** concussion, convergence insufficiency, near point of convergence, vergence facility, positive fusional vergence, vision therapy, mild traumatic brain injuries

## Abstract

**Introduction:**

The CONCUSS clinical trial examined the neural changes associated with office-based vergence/accommodative therapy with movement (OBVAM) in concussion-related convergence insufficiency (CONC-CI).

**Methods:**

The following assessments were collected at baseline and post-OBVAM therapy: activity evoked from a functional MRI vergence oculomotor task, near point of convergence (NPC), positive fusional vergence (PFV), vergence facility (VF), and visual symptoms from a sensorimotor vision exam. Fifty-four CONC-CI participants, diagnosed with persisting concussion symptoms between one- and six-months post-injury, were analyzed in group-level results.

**Results:**

Functional activity in the vergence oculomotor network, specifically the frontal eye fields, supplemental eye fields, parietal eye fields, cerebellar vermis (CV), and visual cortex, increased post-OBVAM compared to baseline assessments. Significant increases in post-OBVAM compared to baseline assessments were observed in the visual cortex (bilateral V3 and right area PH) and the CV, via a paired t-test with family-wise error corrected for multiple comparisons (*p* < 0.05). The pooled baseline and post-OBVAM measures revealed that the bilateral functional activities of V3 and CV were significantly correlated with the NPC, PFV, and VF clinical signs, and the right hemisphere area PH within the visual cortex was significantly correlated with VF (Bonferroni-corrected; *p* < 0.001). To determine whether the CONC-CI post-OBVAM functional brain activity differed from that of the binocularly normal control (BNC) data, an unpaired t-test was performed comparing 46 age-matched BNC datasets with 54 CONC-CI datasets. Significant differences in functional activity between BNC and CONC-CI at post-OBVAM datasets were not observed (*p* > 0.05).

**Discussion:**

Results support that OBVAM improves functional brain activity in CONC-CI correlated with NPC, PFV, and VF.

## Introduction

The Amsterdam Consensus on Concussion in Sport Group defines concussion as a “traumatic brain injury caused by a direct blow to the head, neck, or body resulting in an impulsive force being transmitted to the brain that initiates a neurotransmitter and metabolic cascade with possible axonal injury, blood flow change, and inflammation affecting the brain” (Patricios et al., 2023). The World Health Organization estimates that there are about 56 million concussions worldwide annually, making concussion, or mild traumatic brain injury (mTBI), a prevalent problem worldwide (Dewan et al., 2019; Ahmed et al., 2024), where 25 to 30% of individuals develop persistent post-concussive symptoms (PPCS) (Boutis et al., 2018; Ewing-Cobbs et al., 2018). The economic cost associated with concussion is estimated to be hundreds of billions of dollars in the United States alone, when considering medical costs and productivity losses (Maas et al., 2022). Hence, the prevalence and costs represent a significant clinical challenge following mTBI, particularly among adolescents and young adults, which are coupled with a reduction or modification in activities such as sports and academics (Master et al., 2022).

One phenotype of concussion is visual symptoms, such as blurred vision, diplopia, and photophobia (Craton et al., 2017). These visual symptoms are typically caused by oculomotor and accommodative dysfunction (Master et al., 2016; Mares et al., 2019; Scheiman et al., 2021; Wiecek et al., 2021; Wu et al., 2025). The symptoms are speculated to arise from disruptions in neural circuits responsible for oculomotor control and visual processing. One of the most common oculomotor and visual dysfunctions after a concussion is concussion-related convergence insufficiency (CONC-CI), where a person has reduced binocular coordination in either initiating or maintaining fusion when focusing on near targets (Alvarez et al., 2012; Master et al., 2016; Scheiman et al., 2021). Currently, the common practice in concussion management strategies within standard clinical care includes rest and a gradual return to activity. Yet, a recent study reports that when concussion-related convergence insufficiency (CONC-CI) persists for at least 1 month post-injury, further watchful waiting of an additional 6 weeks results in persistent symptoms and only about 10% remediation for CONC-CI (Alvarez et al., 2025a). While a recent review supports that aerobic exercises, physical activity, and vestibular therapeutic intervention remediate symptoms associated with concussion (Patricios et al., 2023), clinical trial studies have yet to investigate oculomotor rehabilitation using functional brain imaging correlated with clinical signs and symptoms.

In this secondary analysis of data from the CONCUSS clinical trial mentioned above, we established two objectives. The first objective examines potential differences in functional brain activity between CONC-CI post-OBVAM and baseline measurements, with a correlation analysis on changes in convergence function and visual symptoms. The second objective assesses whether the functional brain activity datasets post-OBVAM differ significantly from those of individuals with normal binocular vision. This study tests the hypothesis that OBVAM will increase activation in key regions of the vergence network, including the frontal eye fields (FEF), supplemental eye fields (SEF), parietal eye fields (PEF), oculomotor vermis (OV), and visual cortex (VC), making CONC-CI participants more similar to activation levels seen in binocularly normal controls (BNC).

## Materials and methods

### Participants and design

This study enrolled participants with concussion-related convergence insufficiency (CONC-CI) and binocularly normal controls (BNC) as part of the CONCUSS clinical trial (NCT05262361) (Alvarez et al., 2024, 2025b). Adolescents and young adults aged 11 to 25 years from central and northern New Jersey were recruited into the CONC-CI and BNC groups. For the CONC-CI group, eligibility was determined based on a physician-confirmed diagnosis of concussion (co-author AG) and symptomatic CONC-CI diagnosed by an optometrist (co-author MS), as outlined below. For the BNC group, an optometrist (co-author MS) confirmed the participant had normal binocular vision. The study protocol for the CONC-CI group was approved by the Institutional Review Boards (IRBs) of the New Jersey Institute of Technology (NJIT), Rutgers University–Newark, and Drexel University, with NJIT serving as the IRB of record under the SMART IRB agreement, while the study protocol for the BNC group was approved by the IRBs of NJIT and Rutgers University–Newark in accordance with the Declaration of Helsinki. All adults provided written consent. Adolescents signed an assent with a parent or legal guardian providing written consent.

All participants underwent a comprehensive sensorimotor vision examination to confirm group eligibility by a study-certified optometrist (co-author MS). Diagnostic criteria for symptomatic CONC-CI were based on established clinical trial standards (Scheiman et al., 2005a, 2015; CITT-ART Investigator Group, 2019; Alvarez et al., 2020a) which defined the diagnosis as: (1) a Convergence Insufficiency Symptom Survey (CISS) score greater than or equal to 16 points for pediatric participants or greater than or equal to 21 points for adults, (2) a near point of convergence (NPC) break greater than 6 cm, and (3) positive fusional vergence (PFV) less than 15 prism diopters or failure of Sheard’s criterion (Sheard, 1930). The NPC is defined as the closest distance along the midline where a participant can binocularly maintain single vision, measured from the nasion (Alvarez et al., 2025a). PFV refers to the diopter range over which a participant can maintain clear and single vision along midline while looking at a high acuity target 40 cm from midline with a prism bar of 1Δ, 2Δ to 20Δ in increments of 2Δ, and 5Δ to 45Δ in increments of 5Δ. CISS is a 15-item questionnaire that quantifies visual symptoms on a 5-point Likert scale from 0 (no symptoms) to 4 (severe symptoms), with a score range of 0 to 60. Vergence facility (VF) is defined as the rate at which a target can be seen, single and clear, using a 12Δ base-out prism and then a 3Δ base-in prism, defined as one cycle for a one-minute duration. All participants were required to have normal visual acuity and stereopsis. Exclusion criteria for both the CONC-CI and BNC groups included high refractive error (greater than 7.00 diopters of myopia or greater than 2.00 diopters of hyperopia), anisometropia greater than 1.50 diopters between eyes, a history of retinal pathology, ocular surgery, or inability or unwillingness to complete study procedures. For the BNC group specifically, inclusion required normal binocular vision, and exclusion also included a history of brain injury. Comprehensive inclusion and exclusion criteria, along with detailed descriptions of the techniques and tools for the sensorimotor vision examination, are outlined in the CONCUSS study protocol (Alvarez et al., 2024). Demographic data were collected for all participants and included age, sex, race, ethnicity, athletic status, and self-reported attentional difficulties. For participants in the CONC-CI group, additional clinical history was obtained, including the number of clinician-diagnosed concussions and the time since the most recent concussion.

### Office-based vergence/accommodation therapy with movement

Participants in the CONC-CI group participated in office-based vergence/accommodative therapy with movement (OBVAM), as described in detail in the CONCUSS protocol (Alvarez et al., 2024, 2025b). The intervention consisted of 12 to 16 one-hour sessions conducted twice weekly at clinical sites in central or northern New Jersey, United States. To minimize symptom exacerbation, sessions were scheduled on non-consecutive days. Participants were also assigned home-based exercises, with each session lasting approximately 15 min to be completed three times a week on days when they were not engaging in office-based therapy.

The OBVAM protocol is similar to the therapy used in multiple clinical trials studying convergence insufficiency without head injury (Scheiman et al., 2005a, 2015; CITT-ART Investigator Group, 2019; Alvarez et al., 2020a) and differed by the inclusion of head and body motion while performing visual tasks. The therapy consisted of four progressive phases designed to improve function in the vergence and accommodation systems. Participants progressed through phases using predefined criteria, as assessed in detail in the CONCUSS study design. Within each phase, therapy procedures targeted concussion-related oculomotor deficits, including saccades, pursuits, gross convergence, vergence, and accommodation. These procedures were organized into increasing levels of difficulty. Task complexity was often elevated by incorporating head or body motion, thereby enhancing the challenge and promoting multisensory integration. Following completion of the therapy protocol, participants in the CONC-CI group repeated both the sensorimotor vision examination and the fMRI session.

### Functional MRI data acquisition

FMRI data were acquired using a 3 T PRISMA scanner (Siemens Medical Solutions, Parkway Malvern, PA, United States) at the Rutgers University Brain Imaging Center (RUBIC) in Newark, New Jersey, United States. A 64-channel head and neck coil equipped with an EyeLink-1000-compatible mirror enabled monocular right-eye tracking via the EyeLink-1000 infrared eye-tracking system (SR Research, Kanata, ON, Canada). Participants were positioned with their nasion centered in the head coil to ensure symmetrical vergence presentation. Visual stimuli were viewed via an angled mirror positioned 15 cm from the participant’s nasion, reflecting a screen located 80 cm away, resulting in an effective viewing distance of 95 cm. Stimuli were presented using Psychtoolbox (Brainard, 1997; Niehorster et al., 2020) written in MATLAB, with additional functionality from the EyeLink Toolbox, and were projected at a resolution of 1920 × 1,080 pixels on a 32 cm × 18 cm screen as described in detail previously (Sangoi et al., 2025).

Monocular eye movements were recorded at a sampling rate of 250 Hz and a spatial resolution of 0.25°, allowing for real-time monitoring of oculomotor task performance. The eye tracker was mounted on the projector screen apparatus within the MRI bore. Prior to scanning, a nine-point calibration procedure was conducted using a 3 × 3 visual target matrix grid to convert the raw eye position into degrees of rotation. Calibration was repeated as needed if the participant blinked or failed to maintain fixation on the calibration target, ensuring accuracy with audio feedback provided via intercom. During scanning, the EyeLink system provided a live video feed of the eye image and the eye position trace to the control room in real-time, allowing the operator to verify task adherence.

Each imaging session consisted of the following portions: eye movement calibration, a field map scan, a vergence oculomotor stimulus-induced functional scan, and a high-resolution anatomical scan. After eye movement calibration, a gradient-echo field map was collected in the anterior-to-posterior direction. Functional imaging was performed using a multiband echo planar imaging (EPI) sequence with the following parameters: repetition time (TR) = 720 ms, echo time (TE) = 33 ms, field of view (FOV) = 192 mm, flip angle = 90°, spatial resolution = 3 × 3 × 3 mm, 56 axial slices, and 730 volumes. Anatomical imaging was acquired using a Magnetization Prepared–RApid Gradient Echo (MP-RAGE) sequence (TR = 1900 ms, TE = 2.52 ms, T1 = 900 ms, flip angle = 9°, FOV = 256 mm, spatial resolution = 1 × 1 × 1 mm, 176 slices).

The vergence stimulus consisted of a set of three eccentric squares, designed to stimulate the vergence-related neural substrates, with full experimental details described previously (Sangoi et al., 2025). The stimulus demonstrated good repeatability (Morales et al., 2020a). When fused correctly, the inner squares appeared closer to the participant, providing proximal feedback. A block design was implemented: each vergence block included nine pseudorandom eye movements (2–4°) over 25 s, followed by a 25-s rest block. The stimulus-induced fMRI scan included 10 vergence oculomotor ‘task’ blocks alternated with 11 sustained ‘rest’ blocks, beginning and ending with a rest block. All participants completed training sessions prior to scanning to ensure familiarity with the task and the ability to achieve fusion. Successful task performance was confirmed through verbal reports of perceived depth at training and validated via eye tracking acquired during the scan.

### Data preprocessing and statistical analysis

FMRI data in NIfTI format were first converted to the Brain Imaging Data Structure (BIDS) format (Gorgolewski et al., 2016) and preprocessed using a standardized pipeline implemented in SPM12 (Wellcome Center for Human Neuroimaging, UCL, London, United Kingdom) and used in many studies of the vergence neural system (Morales et al., 2020a,b; Hajebrahimi et al., 2023, 2024; Sangoi et al., 2025).

The anatomical image and the first volume of the functional scan were aligned with the anterior commissure as the origin. For the vergence oculomotor task, motion correction was performed, and the functional images were realigned to the first image of each session. Field map correction was applied to improve spatial homogeneity (Togo et al., 2017). The anatomical scan was bias-corrected, skull-stripped, and segmented into gray matter, white matter, and cerebrospinal fluid using SPM12 segmentation tissue probability maps (threshold = 0.5). Next, functional data were co-registered with the anatomical data. All functional images were normalized to the Montreal Neurological Institute (MNI) standard space with a template of 3 mm voxel size (Wu et al., 2018) using the deformation field maps derived from the segmentation step. Temporal regression was then performed to remove nuisance signals associated with head motion, white matter, and cerebrospinal fluid. A total of 34 nuisance regressors were included: six motion parameters, their squared terms, six temporal derivatives, and their squared terms (24 motion-related variables), along with the first five principal components from both white matter and cerebrospinal fluid (10 physiological variables). Temporal filtering was applied between the frequency bands 0.01 and 0.1 Hz, followed by spatial smoothing using a Gaussian kernel with a full-width at half-maximum (FWHM) of 6 mm. Motion artifacts were assessed using the realignment output. Datasets were excluded from further analysis if the mean framewise displacement exceeded 0.5 mm or if more than 20% of volumes exceeded 2 mm of motion.

Following preprocessing, whole-brain functional activation maps were generated using SPM12. A general linear model (GLM) was applied to each participant’s preprocessed data, incorporating the timing paradigm of the vergence oculomotor task. The task blocks were 25 s in duration, with a repetition time (TR) of 0.72 s. Task onset times were convolved with the canonical hemodynamic response function in SPM to estimate voxel-wise beta weights, referred to hereafter as brain activation.

For group-level analyses, FSL’s randomize tool, a nonparametric permutation-based method (Winkler et al., 2014) was used with 10,000 permutations. To control for multiple comparisons, statistical significance was assessed using threshold-free cluster enhancement (TFCE) with family-wise error (FWE) correction at *p* < 0.05 (Smith and Nichols, 2009). All results were visualized using Analysis of Functional NeuroImages (AFNI) (Cox, 1996) overlaid on Montreal Neurological Institute (MNI) anatomical templates. To determine whether significant differences in functional brain activation were observed between the mean CONC-CI post-OBVAM therapy dataset and the mean BNC dataset, the un-thresholded FWE-corrected *p*-value maps were first assessed in AFNI. Next, the statistical comparisons were performed. One-sample t-tests were conducted separately for the CONC-CI and BNC groups to mitigate the variability from individual datasets on group-level statistics. To assess longitudinal changes in the CONC-CI group, a paired t-test was used to compare baseline and post-OBVAM values. Additionally, to assess the between-group differences in the CONC-CI post-OBVAM versus BNC, activation maps were compared using an unpaired t-test. The paired and unpaired comparisons were masked by the average vergence activation map from the BNC group (Sangoi et al., 2025), due to task-specific sensitivity.

### Clinical success or improved definitions

Using predefined definitions (Alvarez et al., 2025b), the CONCUSS clinical trial used a composite score of NPC and PFV to assess success or improved vision function after OBVAM. Success was defined using the following criteria: (1) normal NPC (<6 cm) AND a decrease of ≥ 4 cm AND (2) normal PFV (met Sheard’s criterion and break value > 15Δ), AND an increase of ≥ 10Δ. Improved was defined when the NPC was normal OR decreased by ≥ 4 cm AND PFV was normal OR increased by ≥ 10Δ. Non-responder was defined as a participant who did not meet the improved criteria.

### Clinical correlation analysis

The clusters of voxels demonstrating statistically significant differences in activation following therapy compared to baseline were used to define the regions of interest (ROIs) for further correlation analysis between activation values and clinical measurements. For each significant ROI, the mean beta weight was calculated by averaging the activation values across all voxels within the cluster.

Clinical measures used for correlation analyses included the following primary and secondary post-OBVAM therapy variables as defined in the CONCUSS clinical trial (Alvarez et al., 2024, 2025a): NPC, PFV, CISS, and VF. The Pearson correlation coefficient was calculated between clinical signs and symptoms and mean functional activation within each ROI, using both the baseline and post-OBVAM therapy datasets in MATLAB. All correlation analyses were corrected for multiple comparisons using the Bonferroni correction.

## Results

### Sensorimotor examination with demographics and eye movements

A total of 64 participants were enrolled in the CONC-CI group who completed the OBVAM therapy. One participant was unable to undergo scanning due to discomfort caused by the MRI from wearing braces. Of the remaining participants, six were excluded because they failed to meet the predefined motion criteria, defined as having more than 20% of volumes exceeding 2 mm of framewise displacement at baseline. Three participants were excluded due to excessive motion during the post-OBVAM therapy scan. As a result, 54 CONC-CI participants (mean age: 17.4 ± 3.3 years; 63% female) were included in the final longitudinal analysis. A total of 51 BNC participants were enrolled. One participant did not complete the protocol due to discomfort in small spaces within the MRI, and four others did not meet the predefined motion analysis criteria, leading to their exclusion from the final analysis. The BNC group-level dataset consisted of 46 participants (19.4 ± 1.7 years, 23 female). For the CONC-CI group, the post-OBVAM group averages were within normal range and were, on average, defined as reaching successful remediation (Alvarez et al., 2025a).

The results of the sensorimotor vision examination are summarized in Table 1 and presented as mean ± standard deviation. Only CONC-CI (N = 54) participants who completed scanning and the motion quality control during the baseline and post-OBVAM therapy imaging scans are included in the analysis, and their sensorimotor vision examination results are summarized below. For comparison, the BNC (*N* = 46) group sensorimotor examination results are also summarized in Table 1. The key metrics from the sensorimotor exam used for the diagnostic criteria for CONC-CI included: NPC, PFV, CISS, and VF. Since there was no significant difference between the sensorimotor clinical exam parameters from 12 to 16 one-hour OBVAM sessions (*p* > 0.1), we pooled the sensorimotor and imaging datasets. Demographics of age, sex, race, ethnicity, and clinical history for the CONC-CI group are summarized in Table 1.

**Table 1:** Sensorimotor vision exam clinical metrics of BNC and CONC-CI at baseline and post-OBVAM therapy.

	BNC N = 46	CONC-CI baseline *N* = 54	CONC-CI post-OBVAM therapy *N* = 54
Sex: Female *n* (%)	23 (50)	34 (63)	
Age at enrollment: Mean years (SD)	19.4 (1.7)	17.4 (3.3)	17.6 (3.2)
Race *n* (%)			
American Indian or Alaskan Native	1 (2)	0	
Asian	17 (37)	4 (7)	
Black or African American	3 (7)	1 (2)	
White	21 (46)	41 (76)	
More than one race	1 (2)	6 (11)	
Other	1 (2)	2 (4)	
Do not wish to report	2 (4)	0	
Ethnicity			
Hispanic or Latino	7 (15)	7 (13)	
Number of concussions *n* (%)			
0	46 (100)	0 (0)	
1		29 (54)	
2		15 (28)	
3 or more		10 (19)	
Time since injury, mean weeks (SD)		8.3 (5.2)	21 (7.7)
Type of injury *n* (%)			
Sports related		31 (57)	
Fall		13 (24)	
Motor vehicle		4 (7)	
Pedestrian struck other		1 (2)	
Struck by or against an object		21 (39)	
Struck by Person		12 (21)	
Other		4 (7)	
Self-reported symptoms, mean (SD)			
CISS (points)	9.8 (4.9)	34.6 (10.8)	14.3 (10.4)
Clinical findings, mean (SD)			
Spherical refractive error right eye (D)	−0.7 (1.7)	−0.5 (1.5)	
Spherical refractive error left eye (D)	−0.7 (1.6)	−0.4 (1.4)	
Exodeviation at distance (∆)	−0.5 (0.8)	−1.0 (1.2)	−1.4 (1.6)
Exodeviation at near (∆)	−1.8 (3.6)	−6.3 (3.4)	−5.1 (3.7)
Near point of convergence break (cm)	3.0 (1.1)	10.4 (3.3)	3.1 (1.3)
Near point of convergence recovery (cm)	4.3 (1.3)	12.8 (3.9)	4.5 (1.8)
Positive fusional vergence blur/break (Δ)	27.8 (9.0)	10.8 (2.8)	30 (10.5)
Negative fusional vergence blur/break (*Δ*)	14.3 (5.6)	10.9 (3.9)	15.9 (4.7)
Vergence facility (cpm) (12∆ BO/3∆ BI)	16.8 (4.8)	9.2 (6.8)	18.2 (6.3)

Δ, prism diopters; cpm, cycles per minute; D, diopters; BO, Base Out; BI, Base In. PFV and NFV are measured as the blur point, or break point if blur is not perceived. Exodeviation is negative, Esodeviation is positive. Results support that CONC-CI post-OBVAM are more similar to BNC sensorimotor results.

**Figure 1:**
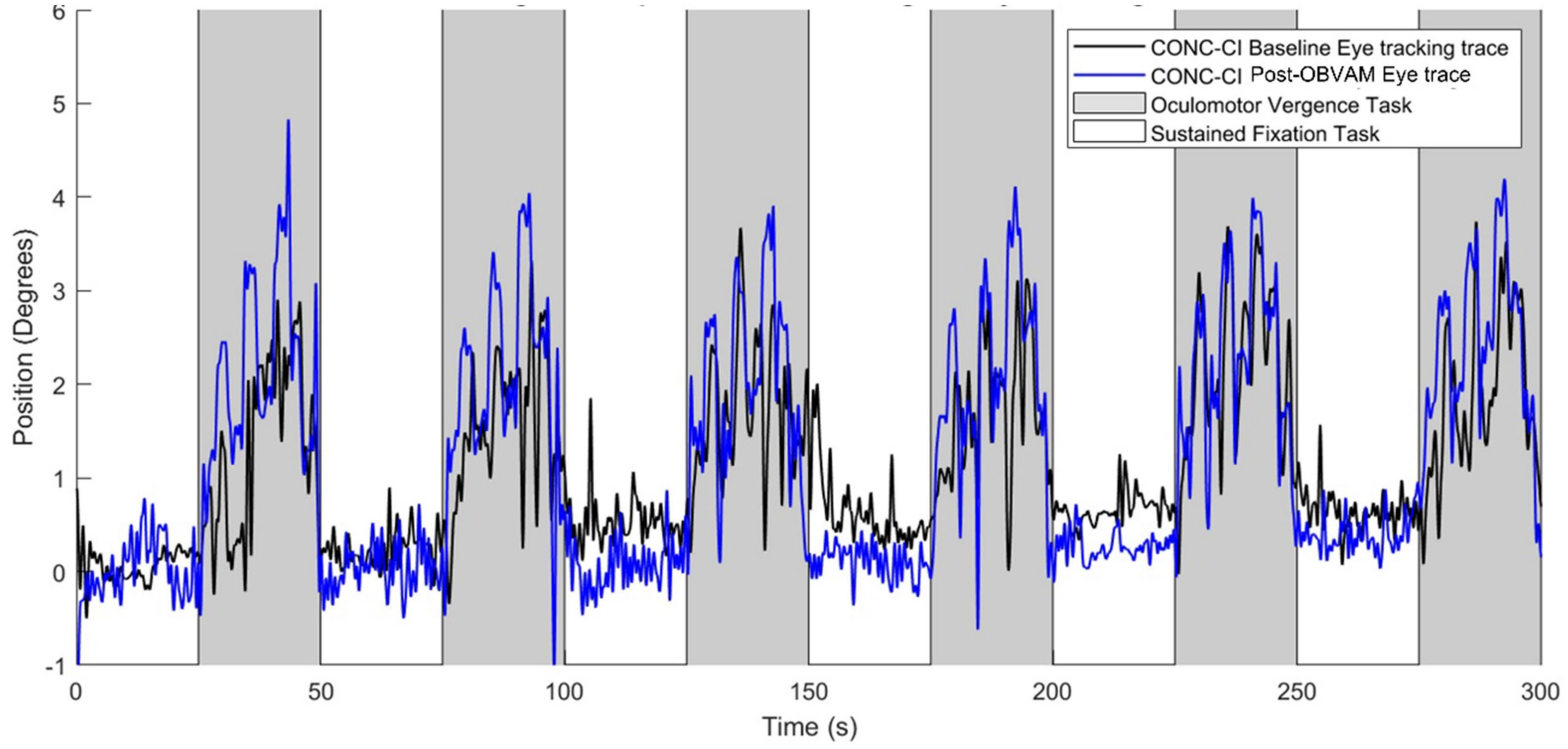
Single participant oculomotor monocular eye tracking during baseline scan (black trace) and post-OBVAM therapy scan (blue trace) from the same individual participant. Eye movement traces are position (in degrees) as a function of time (in seconds). The gray shaded region represents the oculomotor task block, and the white shaded region shows the fixation task block. The post-OBVAM eye position trace (blue trace) supports improved eye tracking compared to the baseline (black trace).

Figure 1 shows the eye movement position traces of an individual participant at baseline (black line) and at the post-OBVAM therapy (blue line) scans. The traces with the white background are from the sustained fixation block (‘off’ or rest task), while the gray background is from the block where the participant is mediating vergence eye movements (‘on’ or active task). The baseline trace shows a reduced amplitude in response to the target vergence demands compared to the post-OBVAM trace.

### Longitudinal functional brain activation

**Figure 2:**
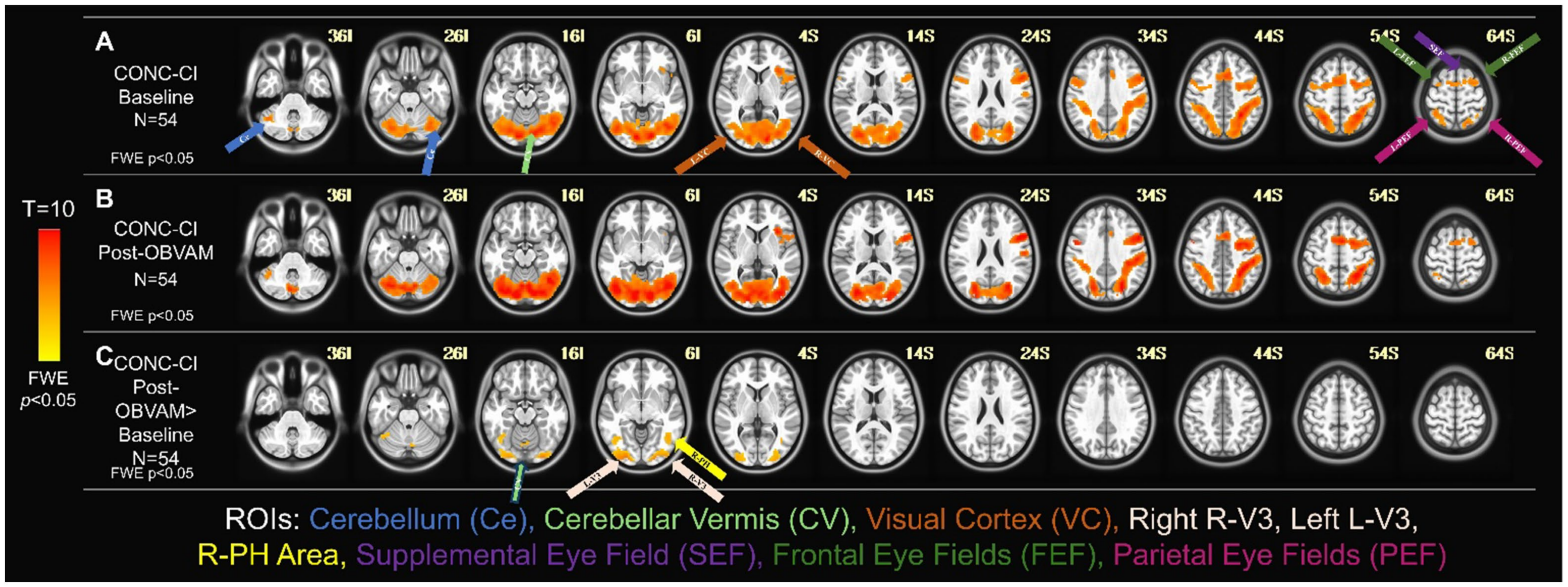
Longitudinal functional activity for CONC-CI group (*N* = 54). One-sample *t*-tests of the CONC-CI group at baseline with TFCE corrected for multiple comparisons at FWE (*p* < 0.05) **(A)** and post-OBVAM therapy **(B)** imaging datasets. The regions show group-level activation in the cerebellum (light blue arrow), cerebellar vermis (light green arrow), visual cortex containing V3 and PH (orange arrow), supplemental eye field (purple arrow), the frontal eye fields (green arrows), and the parietal eye field (pink arrow). **(C)** Paired *t*-test of the CONC-CI at post-OBVAM therapy > CONC-CI at baseline with the BNC mask corrected for multiple comparisons at FWE (*p* < 0.05) shows a significant difference in cerebellar vermis (light green arrow), R-V3 and L-V3 (white arrows), and R-Area Ph (yellow arrow). Following OBVAM, CONC-CI datasets show significantly more functional activity compared to baseline measurements, supporting the conclusion that neuroplasticity has occurred.

Figure 2 shows the average activation maps for the CONC-CI group at baseline (Figure 2A) and post-OBVAM therapy assessment (Figure 2B). Figure 2C presents the results of the paired t-test comparing baseline and post-OBVAM therapy activation within the CONC-CI group, corrected for multiple comparisons using TFCE (*p* < 0.05 FWE-corrected). Four significant clusters were identified and are detailed in Table 2, along with their corresponding peak MNI coordinates. Each cluster is labeled according to the Glasser HCP (2016) multimodal parcellation (Glasser et al., 2016) at the peak location, with additional anatomical localization provided by the AFNI “where am I” tool. The first cluster, with a peak at L (left)-V3, also encompasses V4, Area PH, V3, Fusiform face complex, and V2. The PH area is located within the intersection of the occipital and temporal lobes between the ventrolateral stream and the middle temporal area within the parahippocampal gyrus. The second cluster, with a peak on R (right)-V3, encompasses V4, V3, Posterior InferoTemporal, V1, and V2. The third cluster with a peak on R (right)- Area PH encompasses Area PH, and the area of the fundus of the superior temporal sulcus (FST). The fourth cluster, with a peak on the cerebellar vermis (CV), encompasses CV6, C7, CV7, and Locus Coeruleus (LC)-Crus1. From the sensorimotor exam results and using the predefined criteria of success, improved, or non-responders, post-OBVAM therapy, 41/54 (76%) were classified as successful, 52/54 (96%) were classified as improved, and only 2/54 (4%) were classified as non-responders.

**Table 2:** Clusters of regions of interest (ROI) that were different between the CONC-CI post-OBVAM therapy and the baseline datasets, supporting neuroplasticity, assessed via the significant changes of OBVAM on functional activity.

Number of voxels	X [R]	Y [A]	Z [S]	ROI at peak	ROI
483	−27	−91	−10	L-V3	V4, Area PH, V3, Fusiform face complex, V2
263	30	−91	−1	R-V3	V4, V3, Posterior InferoTemporal, V1, V2
69	45	−55	−4	R- Area PH	Area PH, Area FST
23	3	−67	−19	Cerebellar Vermis (CV)	CV6, C7, CV7, LC-Crus1

**Figure 3:**
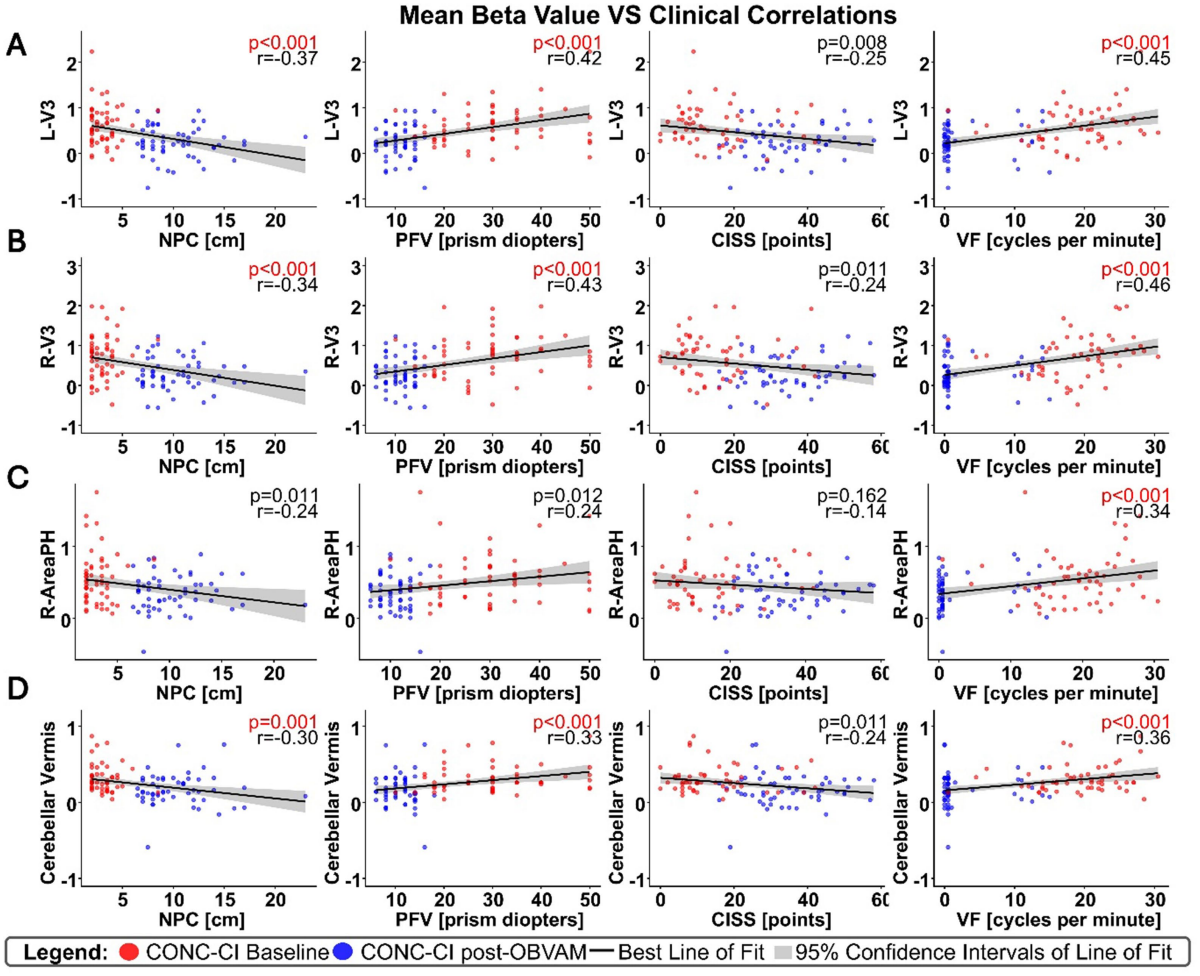
Correlations between significant clusters and sensorimotor clinical exam metrics, with each column as a different clinical metric, and row as a distinct cluster; **(A)** for the L-V3 cluster, **(B)** for the R-V3 cluster, **(C)** for the R-Area PH cluster, and **(D)** for the CV cluster. Correlation results, although not causal, support that several sensorimotor clinical exam metrics are significantly correlated with the beta weights of brain regions that showed significant functional brain activity changes post-OBVAM, indicating the underlying neural mechanism of OBVAM.

For each of the four clusters that showed significant differences between post-OBVAM therapy and baseline in the CONC-CI datasets, mean activation values were extracted at both time points (baseline and post-OBVAM therapy) for each participant and ROI (Table 2). The average values from each ROI (LV3, RV3, R-area PH, and CV) were then correlated with the four parameters from the sensorimotor clinical exam used in the diagnosis of CONC-CI: NPC, PFV, CISS, and VF, resulting in a total of 16 correlations, as shown in Figure 3. A linear regression was used to calculate the best line of fit (solid black line, Figure 3), along with its 95% confidence interval of the best line of fit (shaded gray area in Figure 3). These correlations were corrected for multiple comparisons using the Bonferroni method, where the Pearson correlation coefficient and the corresponding *p*-value are shown in red font if significant, corrected for multiple comparisons. These correlations were corrected for multiple comparisons using the Bonferroni method. These results are presented in Figure 3, with the Pearson correlation coefficient and the corresponding p-value. The correlation analysis is summarized in Table 3. Significant correlations were observed between NPC and PFV with the V3 and cerebellar vermis clusters, while VF showed significant correlation with activation in all four clusters. After correction for multiple comparisons, CISS scores were not significantly correlated to any of the four clusters.

**Table 3:** Pearson correlation coefficient (*r*-values) with *p*-values in parentheses.

ROI	NPC	PFV	CISS	VF
L-V3	**r = −0.37** **(p < 0.001)**	**r = 0.42** **(p < 0.001)**	r = −0.25 (*p* < 0.01)	**r = 0.45** **(p < 0.001)**
R-V3	**r = −0.34** **(p < 0.001)**	**r = 0.43** **(p < 0.001)**	r = −0.24 (p = 0.01)	**r = 0.46** **(*p* < 0.001)**
R- Area PH	r = −0.24 (p = 0.01)	r = 0.24 (p = 0.01)	r = −0.13 (p > 0.1)	**r = 0.34** **(p < 0.001)**
Cerebellar Vermis	**r = −0.30** **(*p* = 0.001)**	**r = 0.33** **(p < 0.001)**	r = −0.24 (*p* = 0.01)	**r = 0.36** **(p < 0.001)**

The significant correlations after Bonferroni Correction for multiple comparisons (*p* < 0.003) are shown in bold font. Results indicate significant correlations between the functional brain activity in regions that showed significant improvement post-OBVAM and the sensorimotor exam results, supporting an underlying neural mechanism of OBVAM.

### Comparison of CONC-CI post-OBVAM therapy to BNC datasets

**Figure 4:**
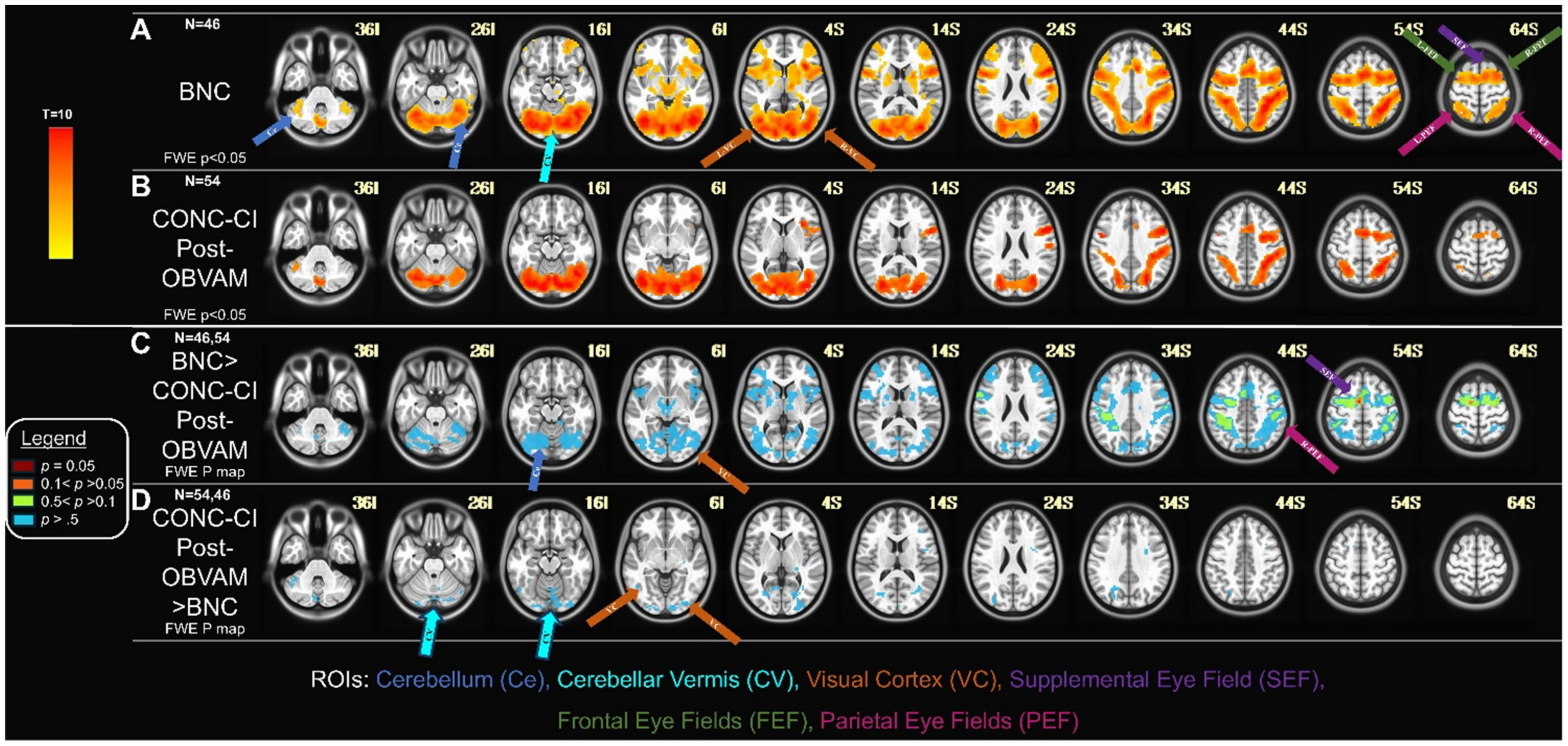
CONC-CI post-OBVAM therapy dataset compared to BNC data: **(A)** Functional brain activity of BNC one-sample t-map with FWE (*p* < 0.05). **(B)** Functional brain activity of CONC-CI post-OBVAM therapy, one-sample t-map with FWE *p* < 0.05. **(C)** Contrast of BNC > CONC-CI post-OBVAM therapy datasets p-map from the FWE corrected two-sample *t*-test. **(D)** Contrast of CONC-CI post-OBVAM therapy > BNC datasets p-map from the FWE corrected two-sample *t*-test. Results show that post-OBVAM, the CONC-CI functional brain activity, is not significantly different from BNC, supporting the notion that OBVAM normalizes brain functional activity.

The second study aim was to determine whether the functional imaging datasets of CONC-CI participants post-OBVAM therapy exhibited functional activation patterns comparable to those of BNC participants. Figures 4A,B display the one-sample t-test activation maps for the BNC group and the CONC-CI group, respectively, post-OBVAM. To assess group-level differences, an unpaired t-test was conducted using FSL’s randomize tool. The unpaired *t*-test investigating whether BNC functional brain activation was greater than CONC-CI post-OBVAM therapy is shown in Figure 4C. Only a single voxel survived TFCE correction at the FWE threshold of *p* < 0.05, located in the supplementary eye field (SEF) at MNI coordinate (6, 4, 53) using the BNC as a mask for activation. Collectively, the CONC-CI post-OBVAM therapy and BNC did not exhibit significant statistical differences in functional brain activity. The parietal eye field (PEF) and frontal eye field (FEF) had *p* > 0.1, while the cerebellum and visual cortex exhibited *p* > 0.5, indicating CONC-CI post-OBVAM therapy exhibited similar functional activity to BNC. The unpaired t-test, investigating whether the functional activity of the CONC-CI post-OBVAM therapy was greater than that of BNC, shows no statistically significant differences, as shown in Figure 4D.

## Discussion

### Neural mechanism of OBVAM therapy

This study is the first to investigate the longitudinal modifications in functional brain activity post-OBVAM therapy in CONC-CI individuals. Four main brain clusters showed significant changes after OBVAM, as compared to baseline functional brain imaging datasets. The peak of each of those clusters is in L-V3, R-V3, R-Area PH, and CV. These findings align with our prior study in CONC-CI, which showed significantly reduced functional brain activation in these same regions compared to BNC (Sangoi et al., 2025), suggesting that OBVAM therapy can facilitate improved functional brain activity. Importantly, these regions play an essential role in visual processing and eye movement functions. First, bilateral L-V3 and R-V3 within the visual cortex are shown to process disparity and motion perception (Nasr et al., 2016; Kennedy et al., 2023), which are visual stimuli cues used to mediate vergence eye movements. Second, R-Area PH is shown to be functionally activated in stereoscopic motion processing (Büchel et al., 1998; Smith and Wall, 2008), which is also an afferent input to the vergence neural circuit. Finally, CV is shown to play a critical role in mediating vergence eye movements in participants with normal binocular vision (Alkan et al., 2011; Morales et al., 2020a; Fogt et al., 2023). Research on the CV has indicated that its role extends beyond normal binocular vision. Reduced CV activity has been observed in convergence insufficiency participants without a history of head injury compared to BNC (Alvarez et al., 2021a), and this activity improves after vergence/accommodative therapy (Alvarez et al., 2019). Collectively, these findings, together with prior investigations of vergence/accommodative therapy for convergence insufficiency without head injury, suggest that the CV is a central hub in the remediation of visual symptoms and improvement of vergence function. Overall, our results demonstrate that OBVAM therapy engages key visual and oculomotor regions, supporting its effectiveness in restoring functional brain activity.

Importantly, the identified regional activations extend beyond imaging findings and translate to clinically meaningful improvements. The functional brain activity post-OBVAM in L-V3, R-V3, R-Area PH, and CV is correlated with VF. VF measures the ability to quickly regain clear binocular vision through a set of prisms (12Δ base-out and 3Δ base-in). Additionally, VF evaluates vergence oculomotor endurance since the test lasts a full minute. A prior review suggests that the VF is abnormally reduced after a concussion compared to those with normal binocular vision (Thiagarajan et al., 2011), and many participants do not naturally recover within 1 year post-injury for VF (Mona-Lisa et al., 2025). Using objective eye movement recording, individuals with convergence insufficiency without head injury have been reported to have slower vergence eye movement speed compared to those with normal binocular vision (Alvarez et al., 2012; Alvarez and Kim, 2013; Scheiman et al., 2019). Objective vergence eye movement recordings in CONC-CI are also reported to be slower than BNC (Alvarez et al., 2021b) and improve after vergence/accommodative therapy (Scheiman et al., 2017). Hence, the decrease in convergence eye movement speed may, in part, be associated with the reduction of functional activity observed in areas of the visual cortex responsible for disparity processing and the CV.

Our results support neuroplasticity of the oculomotor vermis and the disparity processing area of V3 stimulated via OBVAM. OBVAM took place over 12 to 16 one-hour sessions, twice a week, supporting motor learning, as assessed by an improvement in the sensorimotor clinical vision exam, accompanied by an increase in functional activity. OBVAM has additional movement activities that incorporate vergence and accommodative exercises, supporting OBVAM in improving multi-sensory integration. Another finding for OBVAM is that while prior randomized clinical trials on convergence insufficiency without head injury investigated therapy occurring once per week (Scheiman et al., 2005a, 2019; CITT-ART Investigator Group, 2019), CONCUSS supports the notion that therapy twice weekly is well tolerated, which facilitates a return to activities. Given that the functional activity modifications were significantly correlated with the sensorimotor exam measurements, specifically NPC, PFV, and VF, these results support that neuroplasticity occurred, leading to the improved vergence function.

### Comparison to other literature on vergence rehabilitation

The clinical manifestation of CONC-CI and convergence insufficiency in individuals without head injury could be different. CONC-CI is commonly associated with photophobia, dizziness, nausea, and fogginess (Vyas et al., 2025), which are symptoms not commonly observed in individuals with convergence insufficiency who have not experienced a head injury. Substantial evidence-based research within the general population investigating Office-based Vergence and Accommodative therapy (OBVAT) in adolescents (Scheiman et al., 2005a; CITT-ART Investigator Group, 2019) and young adults (Scheiman et al., 2005b; Alvarez et al., 2020b) who have convergence insufficiency without a history of brain injury showed that OBVAT is effective in about 75% of individuals in remediating clinical signs, specifically NPC and PFV, and symptoms assessed via the CISS. These clinical trials were assessed within a Cochrane review (Scheiman et al., 2020), which concludes that OBVAT is an effective treatment for convergence insufficiency without head injury. However, to address the differences in the clinical manifestation of CONC-CI and convergence insufficiency in individuals without head injury, OBVAT is modified to include movement exercises and called Office-Based Vergence/Accommodative therapy with Movement (OBVAM), which is hypothesized to aid in the remediation of dizziness, nausea, and fogginess. For more information, see the CONCUSS clinical trial (NCT05262361) (Alvarez et al., 2024, 2025b).

Because OBVAM builds upon OBVAT, retaining its established therapeutic benefits while incorporating movement-based components to target dizziness, nausea, and fogginess, the prior OBVAT literature provides a crucial framework for interpreting the present findings. Therefore, while not identical, the following section compares results from vergence therapy studies, including OBVAT, with the present OBVAM findings. Furthermore, since no previous longitudinal study has assessed the changes in functional brain activity in CONC-CI participants, previous longitudinal fMRI studies of individuals with convergence insufficiency without head injury could be beneficial in this context. The first fMRI study of vergence therapy on four symptomatic convergence insufficiency individuals without head injury observed increased activation in the cortex and cerebellum after vergence therapy, with activity correlated to sensorimotor vision parameters, specifically NPC and PFV, which is similar to the results reported in the current study (Alvarez et al., 2010). The second study investigated seven convergence insufficiency participants without a history of head injury, with four participants receiving OBVAT and the remaining three receiving placebo/sham therapy (Widmer et al., 2018). This study utilized red/blue glasses to stimulate vergence and suggested that the visual and attentional networks are overstimulated in convergence insufficiency participants without head injury at baseline, and that functional brain activity is reduced after both the active (OBVAT) and sham therapy groups. The results from the study by Widmer et al. and the current study are in contrast. Besides a small sample size, one discussion arising from the study by Widmer et al. was that overstimulation could be due to the increased attention required to learn the task, as both the placebo and active therapy groups showed decreased activation after therapy compared to the baseline imaging datasets. Furthermore, the visual stimuli and the visual presentation sequence are different between the studies. Alvarez et al. (2010) used physical targets that stimulated central/foveal disparity, accommodative, and proximal cues, presented in a pseudo-random order. In contrast, Widmer et al. (2018) employed red/blue random dot stereograms with red/blue glasses, utilizing a large visual field of disparity stimulus within a predictable jump protocol. The current study used eccentric squares that stimulated disparity and proximal vergence in a pseudo-random order. Therefore, there are methodological differences between the studies as mentioned above and the current study. Notably, the visual stimuli and sequences (pseudo-random versus predictable) differ between the studies Alvarez et al. (2010), where predictable stimuli are shown to stimulate the dorsolateral prefrontal cortex more than non-predictable stimuli. There may also be differences in the sustained fixation, which changes convergence peak velocity (Kim et al., 2011a,b). Widmer et al. (2018) is also a pilot study involving a small number of participants. To overcome small sample-sized studies, the CINAPS study on 50 young adults with convergence insufficiency without head injury had half (*N* = 25) participate in OBVAT and the other half participate in sham therapy. At baseline, the functional activity was reduced in the frontal lobe and CV and significantly improved post-OBVAT, but not post-sham therapy (Alvarez et al., 2019). Furthermore, the CINAP study acquired resting-state imaging data to examine the brain’s functional connectivity when participants were not engaged in a specific visual task. Results showed a reduced whole-brain functional connectivity in convergence insufficiency compared to BNC at baseline (Hajebrahimi et al., 2023). Additionally, the functional connectivity between the frontal lobe and cerebellar vermis was significantly strengthened post-OBVAT, correlating with clinical improvements, but not in the sham group (Hajebrahimi et al., 2024), suggesting that OBVAT can alter the underlying functional architecture of the brain. Finally, it is worth noting that the functional activity reported in the current study is significantly correlated with the sensorimotor clinical signs (NPC, PFV, and VF) that are also used in the diagnosis of convergence insufficiency. In summary, comparing our current findings with previous vergence therapy studies investigating convergence insufficiency without head injury, our CONCUSS clinical trial results on CONC-CI support that the visual cortex and the CV are important regions of interest in the rehabilitation of vergence function after a concussion.

### Visual symptoms assessed via CISS

The lack of a significant correlation between visual symptom survey CISS scores and neural activation is not surprising. Other studies examining the effectiveness of therapeutic interventions have found that the CISS is an insensitive tool, easily influenced by comorbid ocular conditions such as dry eye, allergies, and other non-ocular conditions (McGregor, 2014; Phillips, 2017; CITT-ART Investigator Group, 2019). After a concussion, many comorbid ocular and vestibular conditions are present. Additionally, the CISS is 25 years old and was developed before handheld electronics became as widespread as they are today. Despite these limitations, the CISS remains the gold standard and the only validated visual symptom survey for use in an RCT investigating convergence insufficiency. Therefore, to compare our results with previous studies, we used the CISS as an outcome measure.

### Study limitations and future directions

Our team is still collecting one-year follow-up data to assess whether the significant functional activity changes observed here are sustained for one-year post-OBVAM. This finding will be the subject of a subsequent publication. Concussion is a highly variable dysfunction, and the recovery also has high variability (Chamard and Lichtenstein, 2018; Patricios et al., 2023). We chose to improve the homogeneity by studying the CONC-CI population, which accounts for about half of the concussion population with persisting symptoms (Master et al., 2016; Scheiman et al., 2021). The private practice from which we recruited had 63% female participants for the CONC-CI, and the control group of BNV had 50% female participants. The difference in the percentages of sex could be a confounding variable. Future randomized clinical trials should have approximately 50 participants per arm, given the observed variability in concussion, with the inclusion of a control arm. The average age difference between the control group and the CONC-CI group was about 2 years, which, considering the range studied, is not clinically meaningful. Given the heterogeneity of concussions, we chose to study an age range of 11 to 25 years to investigate the adolescent/young adult brain with a nonpresbyopic (less than 35 years) visual system. Studying participants much younger or older could introduce confounding variables and require substantially more age- and sex-matched control participants. Future studies should investigate younger and older participants to determine whether the results observed here generalize to different age groups and investigate the impact of the severity of head injury on the results.

Additionally, due to the importance of specific brain regions associated with CONC-CI and neuroplasticity following rehabilitation, future studies that concentrate on targeting rehabilitation-related brain regions, such as the CV and the portions of the visual cortex involved in disparity processing, are warranted. Future studies could benefit from the use of other therapy modalities, such as virtual reality vision therapy (Yaramothu et al., 2019). Virtual reality vision therapy maintains a fixed focal length and therefore does not directly stimulate accommodation, although it may indirectly stimulate accommodation through accommodative vergence crosslinks (Maxwell et al., 2010; Fine S. N. et al., 2025; Fine S. et al., 2025). OBVAM intentionally stimulates the accommodative system with blur stimuli; hence, it is unclear whether virtual reality vision therapy will produce the same effectiveness or utilize the same neural mechanism as OBVAM. It is also unclear whether VRVT will stimulate more or fewer saccades due to movement in the background (Semmlow et al., 1998, 2009), which may influence therapy effectiveness. Future investigations should also be conducted to examine the number of therapy sessions and assess how therapeutic dosing may impact functional brain activity and sensorimotor clinical examination results.

## Data Availability

The raw data supporting the conclusions of this article will be made available by the authors, without undue reservation.
